# Kallmann syndrome with *FGFR1* and *KAL1* mutations detected during fetal life

**DOI:** 10.1186/s13023-015-0287-9

**Published:** 2015-06-09

**Authors:** Julie Sarfati, Claire Bouvattier, Hélène Bry-Gauillard, Alejandra Cartes, Jérôme Bouligand, Jacques Young

**Affiliations:** Univ Paris-Sud, Le Kremlin Bicêtre, F-94276 France; INSERM UMR-1185, Le Kremlin Bicêtre, F-94276 France; Assistance Publique-Hôpitaux de Paris, Bicêtre Hospital, 78 rue du Général Leclerc, Le Kremlin-Bicêtre, F-94275 France; Department of Reproductive Endocrinology, 78 rue du Général Leclerc, Le Kremlin-Bicêtre, F-94275 France; Molecular Genetics and Hormonology Department, 78 rue du Général Leclerc, Le Kremlin-Bicêtre, F-94275 France; Department of Pediatric Endocrinology, 78 rue du Général Leclerc, Le Kremlin-Bicêtre, F-94275 France

**Keywords:** Congenital hypogonadotropic hypogonadism, Kallmann, Syndactyly, Kidney agenesis, Prenatal diagnosis, FGFR1, KAL1

## Abstract

Kallmann syndrome (KS) patients carrying *FGFR1* mutations can transmit the disorder to their offspring as can asymptomatic female carriers of mutations in *KAL1*. We describe for the first time two cases in which KS was suspected during fetal life because of the family context and malformation detection by fetal ultrasound: syndactyly or unilateral renal agenesis in subjects with respectively *FGFR1* and *KAL1* mutations. In relevant family history, ultrasound monitoring can detect KS associated signs before birth and thus enable neonatal diagnosis and early management. These observations also underline the importance of genetic counselling for patients who may transmit KS to their offspring.

**Electronic supplementary material:**

The online version of this article (doi:10.1186/s13023-015-0287-9) contains supplementary material, which is available to authorized users.

## Introduction

Kallmann syndrome (KS; MIM 308700, 147950,244200, 610628, 612370, 612702) is a rare disease characterized by congenital hypogonadotropic hypogonadism (CHH) and an altered sense of smell in both genders [1–3]. KS results from abnormal neural development affecting both the olfactory tracts and GnRH neuron migration. The genetics of KS is complex [1–3]: more than 15 genes have been linked to the disease, with several modes of transmissions [4–16]. Monogenic forms with X-linked, autosomal dominant and autosomal recessive transmission have been identified [2–7]. Digenic and oligogenic forms with less clear modes of transmission were described more recently [2, 3]. The X-linked form is due to mutations in *KAL1* (Kallmann syndrome 1), the first responsible gene to be discovered [4, 5], presently called *ANOS1* (HUGO nomenclature). In this genetic form, females do not generally develop KS [4, 17] but carry *KAL1* mutation in the heterozygous state [18]. KS patients with *KAL1* mutations may have a variety of associated disorders of a neurological or urogenital nature [2, 19], the most frequent being mirror movements, renal anomalies (unilateral or bilateral agenesis, horseshoe kidney) [20] and neurogenic deafness [2, 19, 21]. The second KS responsible gene to be identified was *FGFR1* (Fibroblast Growth Factor Receptor 1); loss-of-function mutations of this gene cause a form of KS with autosomal dominant transmission [6, 17, 21]. Other disorders frequently associated with this latter form include midline anomalies (cleft lip or palate) [2, 6, 21, 22], skeletal anomalies of the hands or feet [23, 24], dental abnormalities [6, 25], and deafness [6, 21]. Infertility in men and women with KS can be corrected by gonadotropin administration to induce spermatogenesis [26] or ovulation [27, 28]. Given the success of these treatments, there is a growing need for genetic counselling [1, 2, 29, 30]. In addition, early diagnosis of KS would enable more timely management of affected newborns [31].

Herein we report for the first time two cases of KS due to *FGFR1* and *KAL1* mutations respectively in which the diagnosis was identified before birth via the known genetic basis of KS in the parents and non-invasive monitoring (fetal ultrasound) to identify KS-associated phenotypes. Confirmation of KS at birth enabled early management of the two children.

## Case reports

All the participants or parents gave their written informed consent for hormonal, morphological and genetic analyses, performed as part of a non-interventional study, conducted as part of usual patient care. The study was performed in keeping with the provisions of the French Bioethics Law and the Declaration of Helsinki and after approval by the Bicêtre Hospital ethic committee (Comité de protection des personnes Ile de France, Hôpital Bicêtre).

### Family 1

**Fig. 1:**
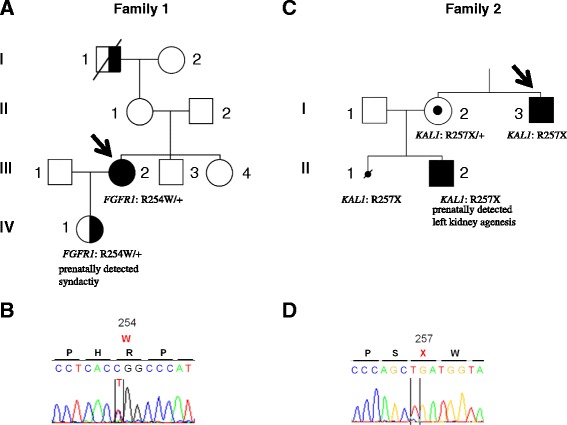
Family pedigrees and corresponding *FGFR1* and *KAL1* mutations. **a** Family carrying the p.R254W (c. 760C > T) *FGFR1* mutation. Squares represent males and circles females. Filled symbols denote the individual with KS (both hypogonadotropic hypogonadism and anosmia/hyposmia). Right half-filled symbols denote individuals with anosmia (subjects I-1 and III-2) and/or olfactory bulb aplasia/hypoplasia (subjects III-2 and IV-1). The propositus (subject III-2) is indicated by an arrow. Subject IV-1 was conceived following ovarian stimulation of the mother (subject III-2) with recombinant human FSH. Sonographic monitoring of the fetus showed signs of Kallmann syndrome (see also Fig. 3 and text). +: wildtype allele. **b** Results of automatic DNA *FGFR1* sequencing encompassing the c. 760C > T heterozygous mutation in the propositus. **c** Family carrying the (c. 769C > T) *KAL1* mutation. The propositus (subject I-3) in this family is indicated by an arrow. The open symbol containing a black dot indicates the unaffected carrier (subject I-2). The small crossed black circle (subject II-1) indicates medical termination of a first pregnancy following the discovery of bilateral kidney agenesis. This fetus carried the same p.R257X *KAL1* mutation. In patient II-2, unilateral kidney agenesis was detected by sonography during fetal life, and *KAL1* analysis at birth confirmed that he also carried the p.R257X *KAL1* mutation in the hemizygous state (see text). **d** Results of automatic DNA *KAL1* sequencing encompassing the hemizygous c. 769C > T mutation in the propositus

The proband of this family (Fig. 1a) was a 32-year-old woman who consulted for infertility. She had breast development at the age of 12.5 years and menarche at 14.5 years. On consulting for oligomenorrhea (4 menstrual bleeds per year) at the age of 18, she was prescribed a combined oral contraceptive, without an etiological work-up, and subsequently had regular menses. Wishing to become pregnant, she stopped using contraception at 29 years of age. Oligomenorrhea recurred and she failed to conceive despite regular intercourse and her partner’s normal sperm count. She then consulted a gynecologist who prescribed her clomiphene citrate. After four rounds of treatment, which led to neither ovulation nor pregnancy, she was referred to our department for an Endocrine consultation.

During the interview she noted no sense of smell (anosmia) that was also present in her maternal grandfather. In addition, she reported poor hearing as well as absent premolars and wisdom teeth (Fig. 1a) [6, 25].

**Fig. 2:**
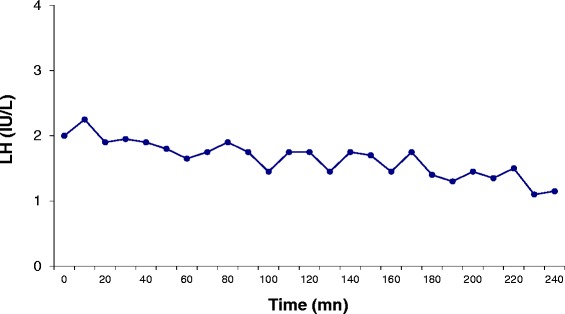
Baseline secretory pattern of LH release in the female subject III-2 of Fig. 1a during 6 h of basal evaluation at 10-min intervals. At the time of evaluation, the patient’s serum estradiol level was 46 pg/ml, and the serum inhibin B level was 32 pg /ml. No LH pulses were detected

Based on the clinical presentation of infertility in the setting of partial pubertal development and congenital anomalies (anosmia, hearing loss, missing teeth) Kallmann syndrome with partial gonadotropin deficiency was suspected [2, 18]. MRI revealed bilateral agenesis of the olfactory bulbs and of the left olfactory tract, as well as a hypoplastic right olfactory tract, reinforcing our suspicions. Olfactometry [32] confirmed her severe hyposmia. Initial hormonal evaluation of the gonadotropic axis showed an estradiol level of 43 pg/mL (N = 25–90). The LH and FSH levels were respectively 2.7 IU/L (normal range in the early follicular phase = 3.0–7.0) and 4.6 IU/L (normal range in the early follicular phase = 2.7–7.0). The inhibin B level was 28 pg/mL (normal range in the early follicular phase = 60—132). A 6-h study of pulsatile LH secretion performed as previously reported [33] showed no significant pulses (Fig. 2). Ovarian MRI showed a right ovarian volume of 2.1 ml (normal volume = 6.4 ± 2.2 ml [34]) with four follicles 2–9 mm in diameter, and a left ovarian volume of 1.9 ml with 6 follicles all smaller than 4 mm.

Other antepituitary functions (free T4: 16.5 pmol/L [11–24, 35], TSH: 0.75 mIU/L (0.3–4.5), peak cortisol (25 μg/dL; N > 18) and peak GH (45 mIU/L; N > 20) in the insulin tolerance test were normal, as was the prolactin (11 ng/mL (N = 10–15). Pituitary MRI showed a normal antepituitary size and a normal pituitary stalk. CT Scan showed normal semicircular canals. Audiometry showed partial bilateral deafness predominating at 2000 Hz.

In view of her family history, deafness and dental agenesis suggestive of autosomal KS [2, 6, 21, 25], *FGFR1* gene analysis was performed revealing a recurrent heterozygous R254W missense mutation that has previously been shown to be loss-of-function in vitro [36].

As she wished to have children, genetic counselling was provided, during which she was informed that there was a theoretical 50 % risk of transmitting the disease to her children [1, 2, 6, 21, 22, 30].

**Fig. 3:**
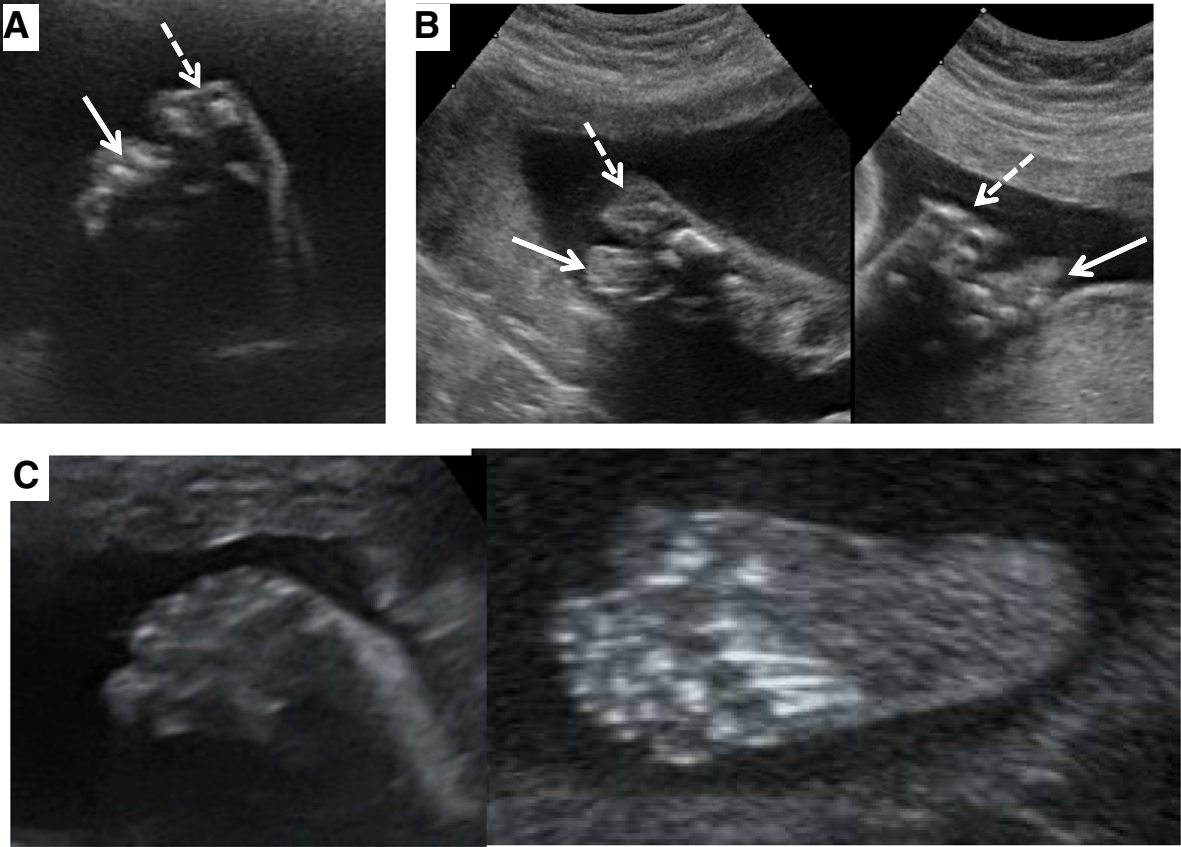
Ultrasound examination of the feet of the fetus with the p.R254W *FGFR1* mutation at 23 weeks of gestation. **a** and **b**: Ultrasound views respectively of the soles of the right and left feet of the fetus carrying the p.R254W FGFR1 mutation. The dashed white arrow shows the syndactyly of toes I and II, and the solid white arrow that of toes III and IV. **c** Plantar ultrasound view of a normal fetal foot

FSH stimulation (75 IU daily, Gonal-F, Merck Serono, Lyon, France) led to the growth of a dominant follicle. Mono-ovulation was triggered with recombinant hCG (Ovitrelle (R), Merck Serono, Lyon, France), and the luteal phase was supported with hCG [27, 28]. She became pregnant, and fetal ultrasound examination performed at 12 weeks showed no abnormalities. A second US examination during the 23rd week of gestation showed a female fetus with bilateral syndactyly (merging of the 1st and 2nd toes and the 3rd and 4th toes) (Fig. 3a and b), an associated sign described in KS with *FGFR1* mutations. The fetus was assumed to have KS due to maternal transmission of the *FGFR1* mutation [2, 6, 24]. Normal delivery took place at the 41st week of gestation. Physical examination of the newborn confirmed the ultrasound anomalies but showed no other malformations. Genetic analysis of a blood sample confirmed the presence of the maternal *FGFR1* R254W mutation in the heterozygous state. MRI performed at the age of six months showed hypoplastic olfactory bulbs bilaterally.

### Family 2

**Fig. 4:**
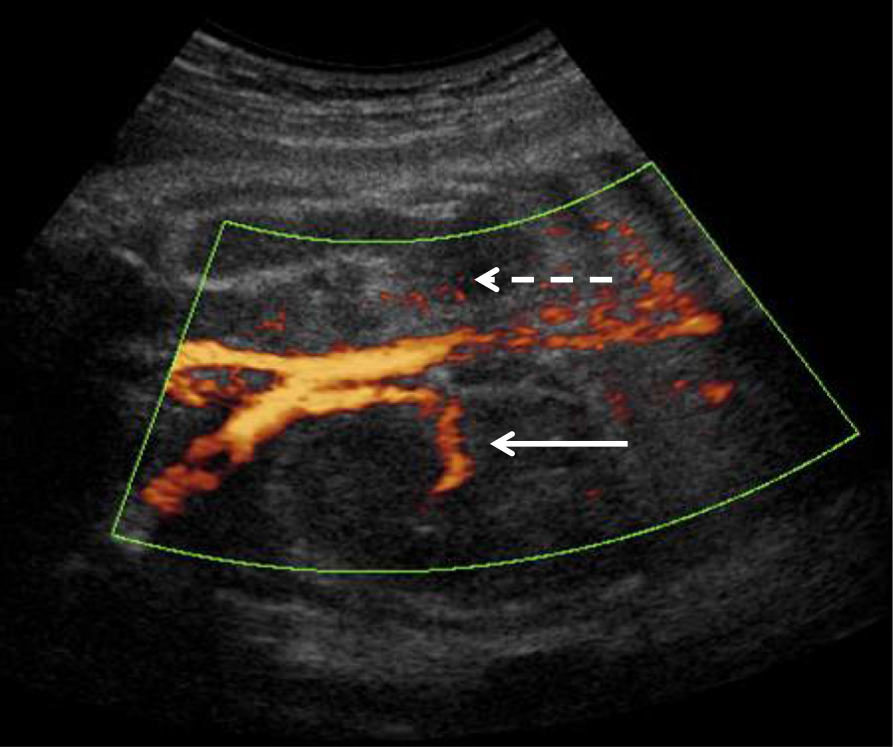
Renal ultrasound at 28 weeks of gestation in the fetus with the p.R257X *KAL1* mutation. The solid white arrow shows the normal right kidney with its pedicle (Doppler US). The dashed white arrow indicates the absence of the left kidney and renal pedicle

The proband was managed in the Endocrinology Department of Bicêtre Hospital, France, for X-linked Kallmann’s syndrome diagnosed at age 17 years (Fig. 1c). Physical examination at diagnosis showed micropenis (<2.5 cm) and bilateral cryptorchidism. Molecular studies revealed a *KAL1* mutation (c.769C > T, p.R257X) consistent with his clinical severity [37]. His sister initially refused to be screened for asymptomatic carrier status of this hemizygous *KAL1* mutation. During her first pregnancy, bilateral renal agenesis was found in her male fetus and she opted for therapeutic termination at 25 weeks [38]. Subsequently, she accepted *KAL1* genetic analysis that confirmed her status as an unaffected carrier of the previously reported *KAL1* mutation (p.R257X) [38]. Ultrasound monitoring of a second pregnancy revealed unilateral (left) renal agenesis in the male fetus (Fig. 4), again pointing to X-linked Kallmann’s syndrome [19, 20, 39].

At delivery, the newborn’s weight and length were normal (weight of 3830 g and a length of 50 cm), but he had micropenis (15 mm;-3SD), descended testes and bilateral testicular hypoplasia (mean testicular volume 0.33 ml (sonography); −3SD). Hormone assays done at age 1 month showed low [31, 40] circulating gonadotropins (FSH: 0.18 (normal range: 0.2–3.5); LH: 0.04 IU/L (0.5–6.5)), testosterone (0.1 (0.5–4.8)) and low testicular peptide levels (inhibin B 24 pg/mL (75–575); AMH 69 ng/mL (80–154)), confirming the diagnosis of congenital hypogonadotrophic hypogonadism [31, 40]. Molecular studies done at birth showed that he carried the same *KAL1* mutation as his mother and maternal uncle. Postnatal sonography follow-up confirmed the left renal agenesis and compensatory right kidney hypertrophy (see Additional file 1: Figure S1), together with bilateral agenesis of the olfactory bulbs on MRI at age 4 months. Synkinesia (mirror movements) were also evident and given the frequent association of *KAL1* mutations and hearing loss, additional evaluation at 1 year of age was conducted revealing deafness in the child [1, 2, 19, 21, 41]. This early diagnosis enabled timely prosthetic treatment to improve the sensory deficit. In addition, in view of the severe reproductive phenotype with micropenis [31], and with approval from our local ethics committee, we proposed to treat the infant with recombinant human gonadotrophins for inducing phallus growth and testicular development [31]. Both parents gave their written consent. Notably, the mother reported a major factor in her decision was her brother’s experience who also had severe KS (cryptorchidism and micropenis) and struggled with sexual disorders and infertility that were not corrected by testosterone treatment or long term combined gonadotropin therapy.

Combination therapy with recombinant human pituitary gonadotrophins (LH and FSH, 75 IU/day each, administered subcutaneously via a pump in order to avoid repeated painful injections) [31, 42] was therefore started when the boy was 1 month old and was continued until the age of 7 months. This treatment was associated with a marked increase in testicle size (from 0.33 to 2.3 ml, at sonography) and penis length (from 15 to 38 mm), both of which became normal for age. Testicular volume, evaluated by sonography, was still normal (0.8 mL) one year after the end of gonadotropin therapy.

## Discussion

We describe two cases of Kallmann syndrome in which the diagnosis was strongly suspected during fetal life, in view of the familial context and ultrasound detection of foot deformities in one case and renal agenesis in the other case. To our knowledge this is the first time that prenatal diagnosis of KS has been achieved with a non invasive method. One old report, by Bick et al. [43], describes prenatal diagnosis of a complex malformative syndrome comprising KS, due to a chromosomal deletion, but the method used was amniocentesis, a far more invasive approach. The family had a contiguous gene syndrome due to deletion of 9.2 megabases of the Xp22 region, which includes the *KAL1*, steroid sulfatase (*STS*) and chondrodysplasia punctata (*CDPX1*) genes. This prenatal diagnosis was based on the familial context and on a highly elevated DHEAS level in amniotic fluid, indicating severe steroid sulfatase deficiency. Autopsy following therapeutic termination revealed, besides a horseshoe kidney, absent olfactory bulbs and a small penis, indicating that the fetus had KS [4, 5, 43].

In both the cases described here, prenatal screening for signs of KS enabled appropriate management to begin at birth. In the family 2 newborn, diagnostic confirmation by hormonal tests and *KAL1* gene analysis enabled us to begin hormone therapy to correct penile and testicular hypotrophy [31, 42]. This early correction of genital hypoplasia is likely to have beneficial consequences for the patient’s sexuality and fertility in adulthood [31, 44–47]. Indeed, previous reports have shown that neonatal combined gonadotropin therapy in patients with CHH/KS and severe reproductive phenotype, diagnosed at birth can have a short term beneficial effect on testicular endocrine function and on genital development particularly by a marked increase in penile length. It is therefore possible that the normalization of penis size in the neonate period will lead, during subsequent postpubertal virilization, to a normal adult penis size and thus avoid the sexual disorders often reported by men with CHH and micropenis [31, 44–47].

Moreover, early knowledge of the genotype prompted us to look for other signs associated with this genetic form; in particular, deafness was detected by means of auditory evoked potentials [48], enabling timely prosthetic treatment. Postnatal investigations also allowed us to reassess, by urogenital imaging, the renal malformation detected during fetal life. This confirmed left kidney agenesis and revealed compensatory right kidney hypertrophy (Additional file 1: Figure S1) that preserved renal function. The same postnatal imaging studies showed that the renal disorders were not accompanied by other urogenital anomalies. Finally, the discovery of renal agenesis in the family 2 fetus suggests that this defect develops at a very early stage and does not result from secondary, postnatal atrophy, contrary to statement of some reports [49].

Similarly, in family 1, following prenatal diagnosis with neonatal confirmation, we were able to seek disorders linked to the relevant *FGFR1* mutations in a timely manner. Brain MRI performed at 6 months showed only agenesis of the olfactory bulbs; no clinically inapparent malformations of the midline were discovered [2, 23, 50]. Hearing tests were normal.

Thus, early diagnosis of KS and knowledge of the genotype in the two cases reported here allowed us to look for signs that might otherwise have been overlooked at birth, potentially delaying the diagnosis until puberty [51]. Indeed, disorders associated with KS are generally discovered only when KS itself is diagnosed, usually at the age of puberty, ruling out timely treatment [51]. Early diagnosis also avoids unnecessary investigations and possible misdiagnosis, and enables replacement therapy to begin at the physiological age of puberty, thereby avoiding the frequent psychological impact of delayed pubertal development [44–47].

These two cases highlight the frequency of direct transmission of KS by patients carrying mutations in *FGFR1* or *KAL1* [1, 3, 6]. Likewise, when KS associated with *KAL1* mutation is diagnosed in a male patient, it is necessary to conduct exhaustive investigations based on family genetic screening for heterozygous healthy carriers [1, 2, 29, 30]. Indeed, given the almost complete penetrance of this phenotype in this X-linked form, a male fetus that inherits the mutated allele from his mother will have a high probability of developing KS. These family studies are also necessary in autosomal dominant forms, as the risk of disease transmission to the offspring is theoretically 50 %. In the case of *FGFR1* mutations, genetic counselling is complicated by the variable penetrance of both cardinal and associated signs, even within the same family [2, 6, 21, 37, 50].

However, even if the mutations in *KAL1* and *FGFR1* are very penetrant, other genes contributing to oligogenic forms may have variable expressivity and incomplete penetrance or contribute to the severity of phenotypes in patients carrying deleterious *KAL1* or *FGFR1* mutations [2, 3]. This is a limitation of the experience reported here because not all Kallmann cases associated with *KAL1* or *FGFR1* mutations will be transmitted as reported herein [2, 3].

One interesting feature of family 1 was the proband’s phenotype. The patient reported having normal pubertal development with menarche at age 14, and said her doctors had not attempted to determine the cause of her oligomenorrhea. In our experience, this is quite a common situation in partial female hypoganodotrophic hypogonadism. Indeed, it is principally the complete form with absent puberty that is taught in medical school and described in textbooks, even though a number of case reports and some studies describe the existence of partial forms of IHH in which breast and pubic hair development are present, despite primary amenorrhea in 95 % of cases [2, 52, 53]. Cases of IHH with oligomenorrhea are far more rarely reported [18, 54]. The case described here stresses the importance of diagnosing these very partial forms of KS before considering medically assisted procreation, given the implications for genetic counselling and the need to closely monitor these pregnancies. Finally, the propositus of family 1 presents a paradigme of cases of female KS patient who are not diagnosed until much later in life. There is a well-reported gender discrepancy between male and female CHH/KS cases with approximately 3–5 males diagnosed for each female case. This may be in fact a bias of ascertainment as females are often started on empiric therapy without a full work-up [2, 18, 51–54].

## Additional file

Additional file 1: Figure S1. Post-natal kidney ultrasound performed in the neonate carrying the p.R257X *KAL1* mutation (see also Fig. 3). **Panel A**: Left posterior fossa view showing the absent left kidney. S: spleen. **Panel B**: Right kidney ultrasound revealing compensatory hypertrophy (dotted line indicate kidney length (65 mm).
