# Supplementary material for: Kallmann syndrome with FGFR1 and KAL1 mutations detected during fetal life
Source: Orphanet J Rare Dis. 2015 Jun 9;10:71. doi: 10.1186/s13023-015-0287-9 (PMC4469106; doi:10.1186/s13023-015-0287-9)
Supplement: Additional file 1: Figure S1. — Post-natal kidney ultrasound performed in the neonate carrying the p.R257X KAL1 mutation (see also Fig. 3). Panel A: Left posterior fossa view showing the absent left kidney. S: spleen. Panel B: Right kidney ultrasound revealing compensatory hypertrophy (dotted line indicate kidney length (65 mm). [file 13023_2015_287_MOESM1_ESM.ppt]

## Slide 1
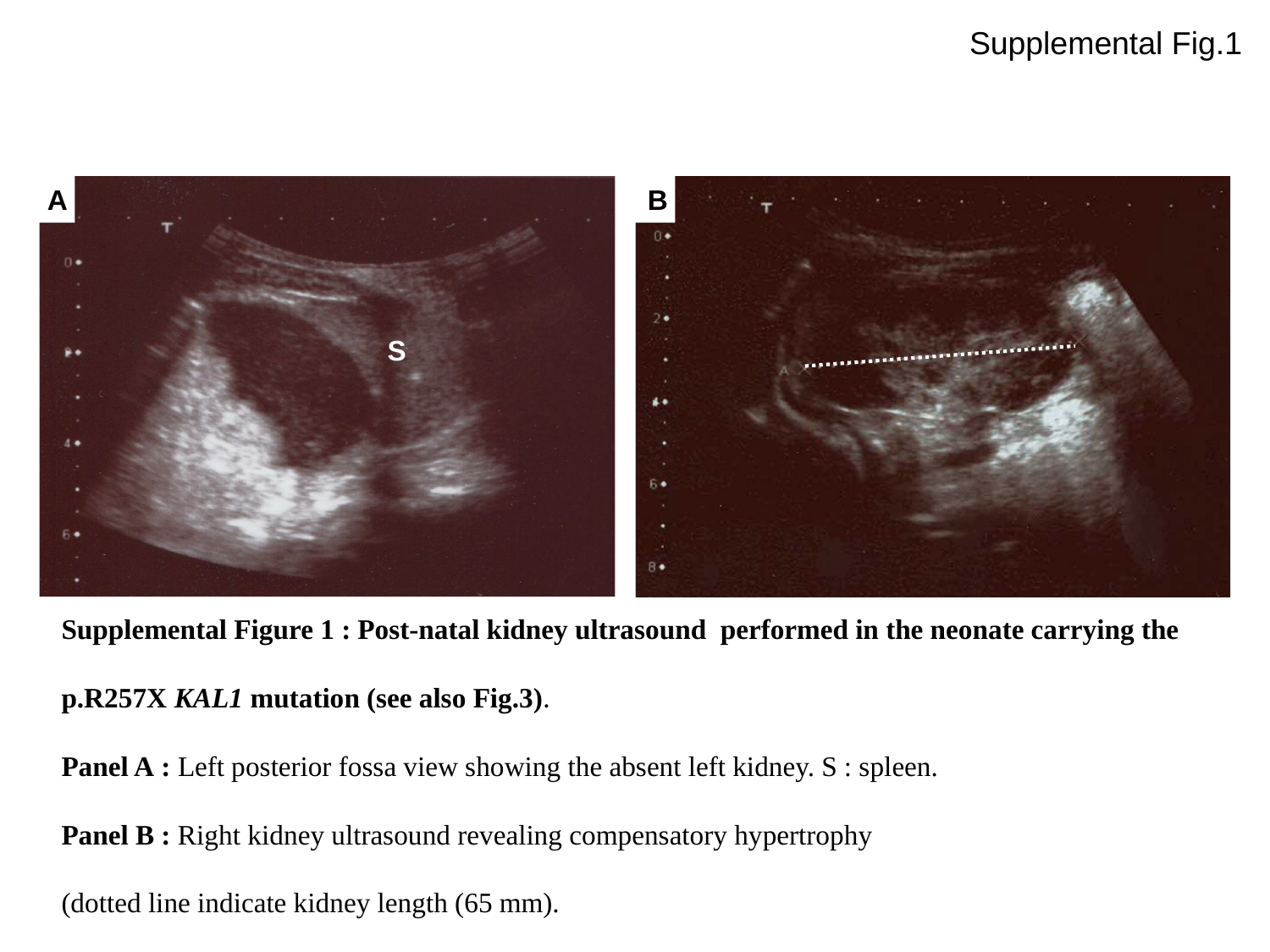

Supplemental Fig.1
A
B
S
Supplemental Figure 1 : Post-natal kidney ultrasound performed in the neonate carrying the
p.R257X KAL1 mutation (see also Fig.3).
Panel A : Left posterior fossa view showing the absent left kidney. S : spleen.
Panel B : Right kidney ultrasound revealing compensatory hypertrophy
(dotted line indicate kidney length (65 mm).
